# Quantitative CT Morphometrics: A Novel Approach for Predicting the Bladder Cancer Grade

**DOI:** 10.7759/cureus.63427

**Published:** 2024-06-28

**Authors:** Tolga Eroglu, Hikmet Köseoğlu, Uğur Yücetaş, Emre Ari, Mustafa Kadihasanoglu

**Affiliations:** 1 Urology, Antalya City Hospital, Antalya, TUR; 2 Urology, Health Sciences University, Taksim Training and Research Hospital, Istanbul, TUR; 3 Urology, Health Sciences University, Istanbul Training and Research Hospital, Istanbul, TUR; 4 Urology, Istanbul University-Cerrahpasa, Cerrahpasa Medical School, Istanbul, TUR; 5 Urology, Istanbul Training and Research Hospital, Istanbul, TUR

**Keywords:** tumor stage, urography, tumor grade, computerized tomography, non-muscle invasive bladder cancer

## Abstract

Background and objective

Bladder cancer (BC) is a common urothelial neoplasm, with non-muscle invasive forms comprising about 75% of cases and generally having better outcomes than muscle-invasive types. Accurate preoperative grading and staging of BC are essential for appropriate treatment planning. This study investigates the efficacy of computerized tomography (CT) in correlating the morphological features of tumors to predict the histopathological grades of BC.

Materials and methods

This retrospective cohort involved 100 patients diagnosed with non-muscle invasive BC, who underwent transurethral resection of bladder tumor (TUR-BT) between January 2010 and August 2021. CT imaging, utilizing a 128-slice CT scanner, was employed to measure the tumor height (H) and contact length (CL). The study considered morphometric parameters across axial, coronal, and sagittal planes. Statistical analyses were conducted, comparing radiological findings with histopathological evaluations. Tumor grading was determined according to the 2004/2016 WHO classification.

Results

Among the 100 patients with primary bladder tumors, 15 were female and 85 were male, with a mean age of 65.28 ± 7.11 years. Furthermore, 58 had high-grade bladder tumors, while 42 had low-grade bladder tumors. Across all planes, high-grade tumors exhibited higher values for the tumor H, CL, and the tumor height-to-contact length (H/CL) ratio compared to low-grade tumors (p<0.05). Notably, the specificity, sensitivity, and diagnostic accuracy of the tumor CL were higher than those of the tumor H and the tumor H/CL ratio. A tumor CL exceeding 19.1mm measured in the axial plane demonstrated 83% sensitivity and specificity for high-grade tumors.

Conclusion

The measured CL of the tumor in the axial plane on computerized tomography urography has high sensitivity and specificity in detecting high-grade tumors.

## Introduction

Bladder cancer (BC) is a prevailing urothelial neoplasia, ranking sixth in global male malignancy incidence and 10th when considering both genders [1]. The age-standardized incidence rates are 2.4 and 9.5 per 100,000 persons per year for women and men, respectively [1]. BC claims the 14th position globally in cancer-specific mortality, with age-standardized death rates of 0.86 and 3.3 for women and men, respectively [1].

BC predominantly originates from urothelium, with approximately 90% stemming from this cellular milieu. Notably, 75% of these cases manifest as non-muscle-invasive Ta and T1 tumors confined to the mucosa [2]. While these non-muscle-invasive tumors exhibit comparatively lower cancer-specific mortality than their muscle-invasive counterparts (T2-4 tumors) [3], the meticulous determination of tumor depth remains pivotal for accurate risk stratification and informed therapeutic decision-making. Non-muscle-invasive bladder cancers (NMIBC) are managed with intravesical agents and transurethral resection of bladder tumor (TUR-BT), while muscle-invasive bladder cancer (MIBC) cases may necessitate a spectrum of interventions, including chemotherapy, radiotherapy, or cystectomy [4].

Accurate BC staging and grading necessitate a multidimensional approach, integrating clinical, radiological, and histopathological data. Advanced imaging modalities and nomograms are instrumental in optimizing preoperative assessments. Local spread of the bladder tumor, lymph node involvement and distant metastases can be detected using radiodiagnostic modalities. Computerized tomography (CT) emerges as a cost-effective, widely available, non-invasive diagnostic method, facilitating the identification of tumors causing urinary system filling defects [5].

Our research endeavors into the morphological aspects, specifically tumor height (H), contact length (CL), and the height-to-contact length (H/CL) ratio-aiming to elucidate their potential impact on the histopathological grade of bladder tumor specimens acquired through TUR-BT. This investigation seeks to contribute insights into BC progression, furthering our understanding of prognostic and therapeutic paradigms.

## Materials and methods

This study received approval from the Health Sciences University Istanbul Health Practice and Research Center Clinical Research and Ethics Committee (Decision No: 2901, dated 13.08.2021). In this retrospective cohort study, 100 patients diagnosed with NMIBC histopathologically and who underwent TUR-BT for primary bladder tumors at our clinic between January 2010 and August 2021 were included. Inclusion criteria comprised male and female individuals aged 18 or older with preoperative CT images up to three months prior to surgery. Of the patients, 15 were female and 85 were male. Only papillary tumors were considered for inclusion. Among the inclusion criteria were diagnosis of primary bladder tumors, no history of intervention prior to CT, and tumors visible on CT. Exclusion criteria encompassed tumors other than urothelial carcinoma, muscle-invasive bladder tumors, insufficient bladder filling on CT, intraepithelial carcinomas, multiple tumors, and metastatic disease. Only the longest tumor measurements were included in the statistical analysis in each case.

The study utilized 128-slice CT imaging (Philips Brilliance, Philips Medical Systems Europe, The Netherlands) with features including 0.5/0.3mm collimation, 150 mAs, 120 kV, 240mm FOV, pitch 0.75, and a 512 matrix. The obtained images were processed on the Philips IntelliSpace Workstation (Philips, Netherlands) and the measurements were made separately by the urologist and radiologist. If measurement results were reported differently among the physicians, they were re-evaluated for proofreading. Measurements of the tumor height (H) and contact length (CL) were conducted in axial, coronal, and sagittal planes during CT examinations. The H/CL ratio was then calculated. The CL was defined as the diameter of the tumor base in contact with the distended bladder wall, while H was considered the distance from the base of the tumor protruding to the lumen to its most distal end.

Radiological results and histopathological findings of the cases were meticulously recorded throughout the study. Tumor stages and grades were based on the 2004/2016 WHO classification and the TNM system updated in 2017 [6] facilitated patient categorization into low and high-grade groups. Subsequently, CT results were compared with postoperative histopathological evaluations. Furthermore, tumor H, CL, and H/CL measurements were compared with the tumor grade.

 IBM SPSS Statistics for Windows, Version 18 (Released 2009; IBM Corp., Armonk, New York, United States) was used for statistical analysis. The Shapiro-Wilk test was used to evaluate whether the data fit the normal distribution. Data that fit the normal distribution were presented as the mean ± standard deviation and data that did not fit were presented as the median (interquartile range). Student’s t-test was used for the analysis of quantitative data with normal distribution, and the Mann-Whitney-U test was used for the analysis of quantitative data without normal distribution. Chi-square or Fisher tests were used for the analysis of quantitative data. Receiver-operating characteristics (ROCs) were calculated with the area under the curve (AUC) for the cut-off value of the data for high-grade bladder tumors. p≤0.05 was accepted as statistically significant in the comparisons.

## Results

In this study, the mean standard deviation for age was calculated to be 65.28 ± 7.11 years, reflecting the cohort’s demographic profile. The average time interval between the CT scan and the primary TUR-BT operation was determined to be 12.6 days.

Upon examination of the histopathological data, 58 patients exhibited high-grade tumors, while 42 presented with low-grade tumors. Additionally, 71 patients were categorized in the Ta stage, and 29 were in the T1 stage, elucidating the distribution across pathological stages. The morphological parameters, including H, CL, and the H/CL ratio, are comprehensively outlined in Table 1 based on the CT images.

**Table 1:** Radiological data of all cases. *: Median (interquartile range)

Parameter	Axial (n=100)	Coronal (n=100)	Sagittal (n=100)
The Height of the Tumor Protruding Into the Bladder Lumen (H)*	17.95mm (17.59)	15.9mm (15.35)	16mm (15.6)
Tumor Contact Length (CL)*	23.9mm (25)	18.55mm (19.05)	18.55mm (22.2)
Height-to-Contact Length Ratio (H/CL)*	0.75 (0.52)	0.85 (0.51)	0.89 (0.54)

Upon scrutinizing the data in relation to tumor grade, it was observed that patients with low-grade and high-grade tumors were comparable in terms of age (64.26 ± 1 vs. 66.01 ± 0.98; p=0.22). However, radiological parameters revealed distinctions, indicating that patients with high-grade tumors consistently manifested higher values across all parameters, as detailed in Table 2.

**Table 2:** Comparison of groups. a: axial plane; c: coronal plane; s: sagittal plane

	High Grade	Low Grade	p
N	58	42	
Tumor Height^ a^	21.8mm (18.3)	13.5mm (13.1)	0.001
Contact Length^a^	36.6mm (26.4)	13.4mm (8.4)	<0.0001
Height-to-Contact Length Ratio^a^	0.58 (0.38)	1.04 (0.94)	<0.0001
Tumor Height^ c^	17.5mm (16.8)	12.9mm (12.5)	0.028
Contact Length^ c^	26.5mm (20)	12.9mm (8.9)	<0.0001
Height to Contact Length Ratio^c^	0.73 (0.4)	1.05 (0.53)	<0.0001
Tumor Height^ s^	17.6mm (16.9)	14.8mm (12.6)	0.018
Contact Length^s^	30mm (19.2)	11.2mm (8.2)	<0.0001
Height-to-Contact Length Ratio^s^	0.79 (0.48)	1.12 (0.64)	<0.0001

To further elucidate the diagnostic utility of the radiological parameters, sensitivity, specificity, positive predictive value, negative predictive value, and overall accuracy were meticulously assessed and are presented in Table 3. Notably, the axial plane measurement of the CL emerged as particularly significant, demonstrating the highest specificity, sensitivity, negative predictive value, positive predictive value, and overall accuracy. In the ROC analysis, the CL of the tumor in the axial plane exhibited the highest AUC value, reaching 0.874. Establishing a cut-off at values above 19.1mm demonstrated an 83% sensitivity and specificity in detecting high-grade bladder tumors, emphasizing its potential as a clinically relevant diagnostic threshold.

**Table 3:** Diagnostic efficacy of radiological parameters. a: axial plane; c: coronal plane; s: sagittal plane

Parameter	Specificity	Sensitivity	NPV	PPV	Accuracy
Height^a^	67%	74%	69%	72%	70.5%
Contact Length^a^	83%	83%	83%	83%	83%
Height^ c^	67%	66%	66%	66%	66.5%
Contact Length^c^	72%	79%	74%	77%	75.5%
Height^s^	60%	60%	60%	60%	60%
Contact Length^s^	74%	81%	80%	76%	77.5%

Case samples

**Figure 1:**
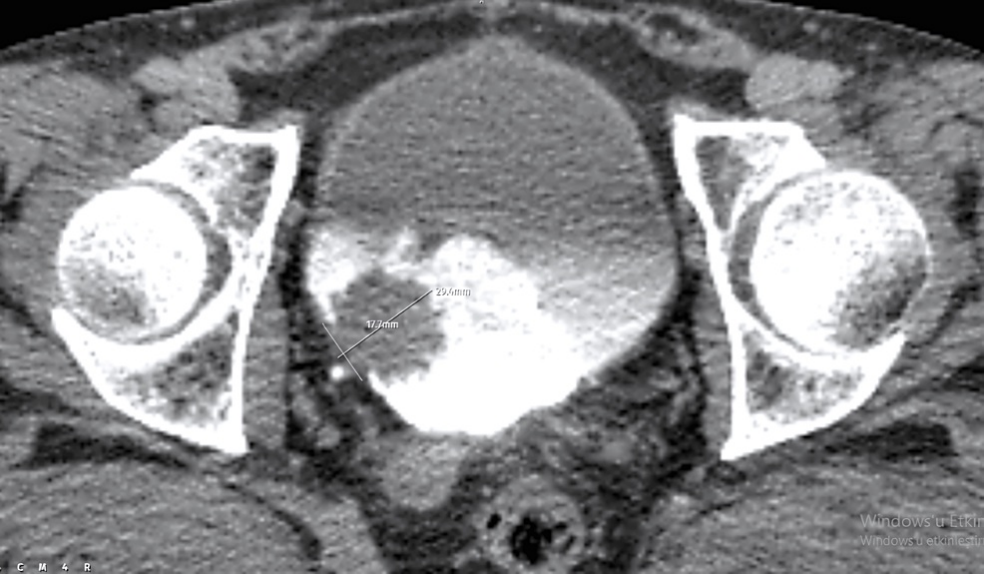
Axial CT section of the bladder tumor in a 56-year-old male patient.

In a 56-year-old male patient with a complaint of hematuria, a papillary mass of 29.4mm in height and 17.7mm in the CL was observed on the right lateral wall of the bladder in the CT examination (Figure 1). The tumor H/CL ratio was calculated as 1.66. Histopathological evaluation of TUR-BT specimens revealed low-grade transitional carcinoma.

**Figure 2:**
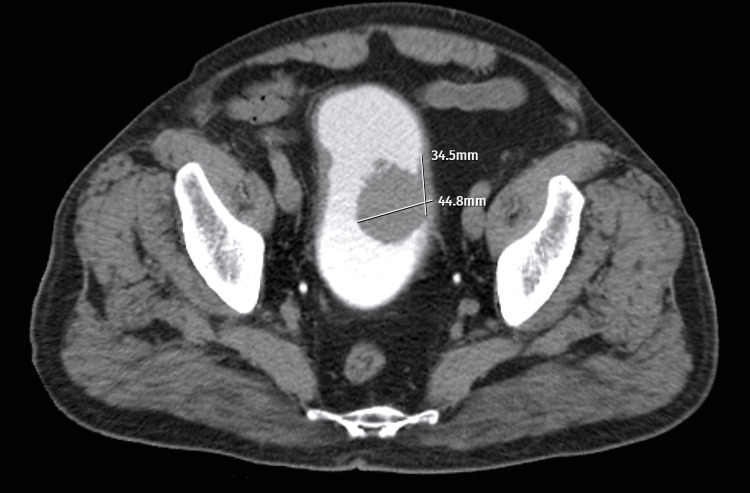
Axial CT section of the bladder tumor in a 69-year-old male patient.

CT examination performed on a 69-year-old male patient with complaints of hematuria and dysuria revealed a papillary mass of 44.8mm in height and 34.5mm in the CL on the left lateral wall of the bladder (Figure 2). The tumor H/CL ratio was calculated as 1.30. In the histopathological evaluation of the TUR-BT specimens, pathology was consistent with high-grade transitional carcinoma.

**Figure 3:**
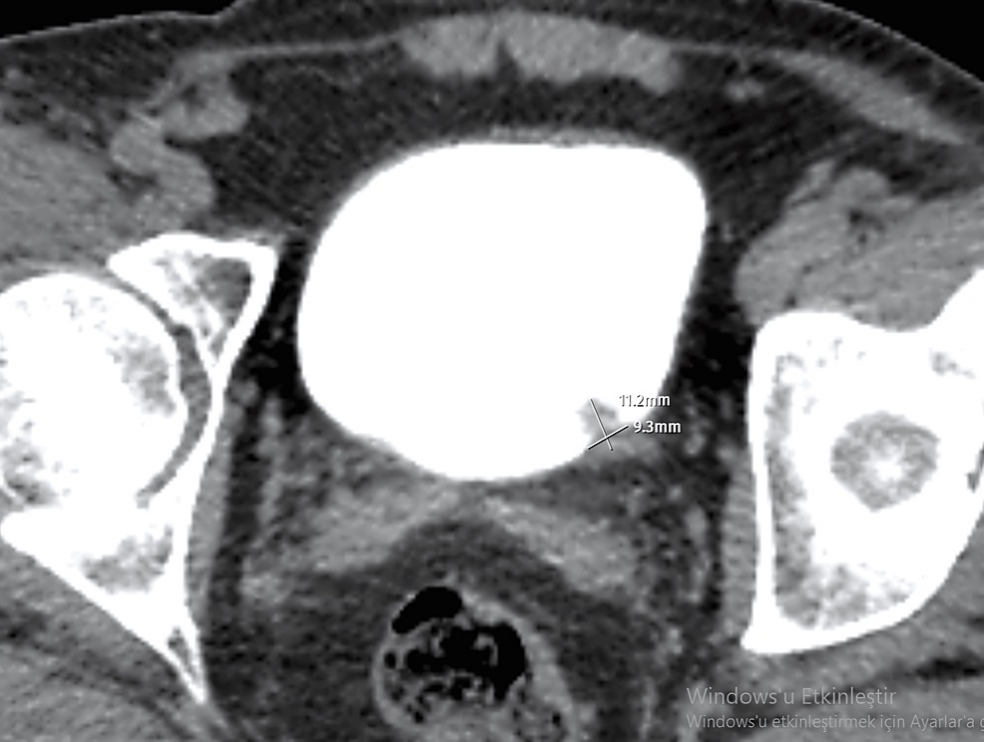
Axial CT section of the bladder tumor in a 63-year-old male patient.

CT examination performed on a 63-year-old male patient with a complaint of hematuria revealed a papillary mass of 11.2mm in height and 9.3mm in contact length at the junction of the posterior wall and left lateral wall of the bladder (Figure 3). The tumor H/CL ratio was calculated as 1.20. In the histopathological evaluation of TUR-BT specimens, pathology was consistent with low-grade transitional carcinoma.

## Discussion

The grade and stage of the tumor are crucial factors in determining the appropriate management approach for patients diagnosed with NMIBC [7]. In 1998, the International Society of Urological Pathology (ISUP) convened a consensus conference where they introduced a novel grading system intended to replace the WHO 1973 system, this updated recommendation was later adopted and included in the WHO classification of bladder tumors in 2004 [7]. The development of this new classification system stemmed from the necessity to create a universally accepted framework for categorizing bladder neoplasms that could be effectively utilized by pathologists, urologists, and oncologists. This classification system addressed not only neoplastic conditions but also standardized the terminology used for preneoplastic lesions [8]. Transurethral ultrasonography has been considered for bladder cancer staging, despite facing technical challenges like acoustic shadows from tumor interface calcification and ultrasound absorption by larger tumors [9]. The depth of bladder wall infiltration is indicated by deep biopsy, although inaccuracies tend to increase as the disease progresses [10]. By determining the depth of invasion, it is possible to assess whether transurethral resection of tumors will be sufficient or if segmental resection or cystectomy will be necessary [11]. Non-muscle-invasive superficial tumors (Ta and T1 tumors) are typically managed with local endoscopic resection followed by adjuvant intravesical installation [12]. While low-grade BCs may find adequate management with intravesical agents and TUR-BT, high-grade tumors, prone to progression and metastasis, may require more aggressive interventions, including chemotherapy, radiotherapy, or even cystectomy [13]. In addition to distinguishing between non-muscle invasive tumors (Ta and T1) and muscle-invasive ones (T2 and T3), it is crucial to identify patients with muscle-invasive cancers who would benefit from radical surgery [8]. Extravesical invasion of bladder cancer (T3 or higher) is primarily assessed using cross-sectional imaging modalities such as CT and MRI. CT is highly advantageous due to its capability to differentiate tumors that are limited to the bladder wall from those that extend into the perivesical fat [8].

BC characterized by its tendency for recurrence and invasive potential necessitates early diagnosis and vigilant monitoring [14]. Precision in staging and grading is imperative for formulating optimal treatment plans in BC. The meticulous determination of accurate staging and grading not only mitigates the risk of unnecessary surgical interventions but also ensures that patients undergo comprehensive and tailored therapeutic interventions. A pivotal consideration in devising an effective treatment strategy revolves around discerning the nature of the tumor-whether it manifests as superficial or infiltrative-and further classifying it as either low or high grade [14].

Cystoscopy stands as the gold standard in diagnosing bladder tumors, given its unparalleled precision [15]. However, the inherent invasiveness of cystoscopy, coupled with potential risks including perforation, stricture, and infection, underscores the imperative need for a non-invasive imaging modality that rivals cystoscopy in reliability. This requirement is particularly crucial for the comprehensive diagnosis, staging, and grading of BC. The quest for a non-invasive alternative aligns with the imperative to mitigate the associated risks while ensuring an accurate and thorough assessment of BC characteristics. In this context, CT stands out as a fast, easily accessible, and non-invasive option, particularly with advancements in CT detector systems enhancing spatial resolution [16].

In a study conducted by Ukimura et al., it was demonstrated that CL of prostatic tumors with the fibromuscular rim could predict extraprostatic extension [17]. They found a significant correlation between CL and extraprostatic extension, achieving an accuracy of 77% in predicting this extension using ROC curve analysis. The authors concluded that the length of tumor contact with the rim was more strongly associated with extraprostatic extension compared to other parameters studied. 

To ascertain if a comparable association exists for bladder tumors, we examined the utility of H, CL, and H/CL ratio in preoperative grading of bladder carcinoma using CT imaging. Our findings indicate that an increase in the CL of the tumor with the bladder wall strongly correlates with a higher grade of the tumor. In our study, a comprehensive analysis of morphometric parameters, including tumor H, CL, and the H/CL ratio, was conducted across axial, coronal, and sagittal planes of pre-operative abdominopelvic CT urography images from patients undergoing primary bladder tumor resection. The statistical findings indicated a significant elevation in these parameters among patients diagnosed with high-grade bladder cancer. Specifically, patients with high-grade tumors exhibited notably longer tumor CL, particularly in coronal sections (26.5 (20) vs. 12.9 (8.9), p<0.0001), and extended tumor CL in sagittal sections (30 (19.2) vs. 11.2 (8.2), p<0.0001) compared to those with low-grade tumors.

In a study conducted by Özden et al., the impact of tumor H, CL, and the H/CL ratio on the staging of BC was explored [18]. The study encompassed 57 cases, and these parameters were systematically assessed in the suprapubic region using ultrasonographic imaging in the transverse plane. Comparative analyses were then performed between the measurements and postoperative histopathological results. Notably, the study revealed a positive correlation between the pathological stage and the contact length of the tumor (p<0.001). The examination of measurements across the axial, coronal, and sagittal planes unearthed statistically significant differences between T1 and Ta tumors (axial: 45.59±4.34 vs. 22.95±2.18, p<0.0001; coronal: 30.81±2.52 vs. 18.52±1.3, p<0.00001; sagittal: 35.05±2.73 vs. 17.76±1.42, p<0.0001). There was no statistically significant disparity was observed in the tumor height concerning the stage of the tumor (p>0.05). Furthermore, the study elucidated that the tumor height in the axial plane, as measured by ultrasonographic imaging in the transverse plane, exhibited no discernible differences across stages in the measurements obtained with CT (29.23±2.73 vs. 18.9±1.28, p=0.301). In our study, patients with the T1 stage demonstrated higher values in all parameters compared to those with the Ta stage, with the exception of H in axial sections.

**Figure 4:**
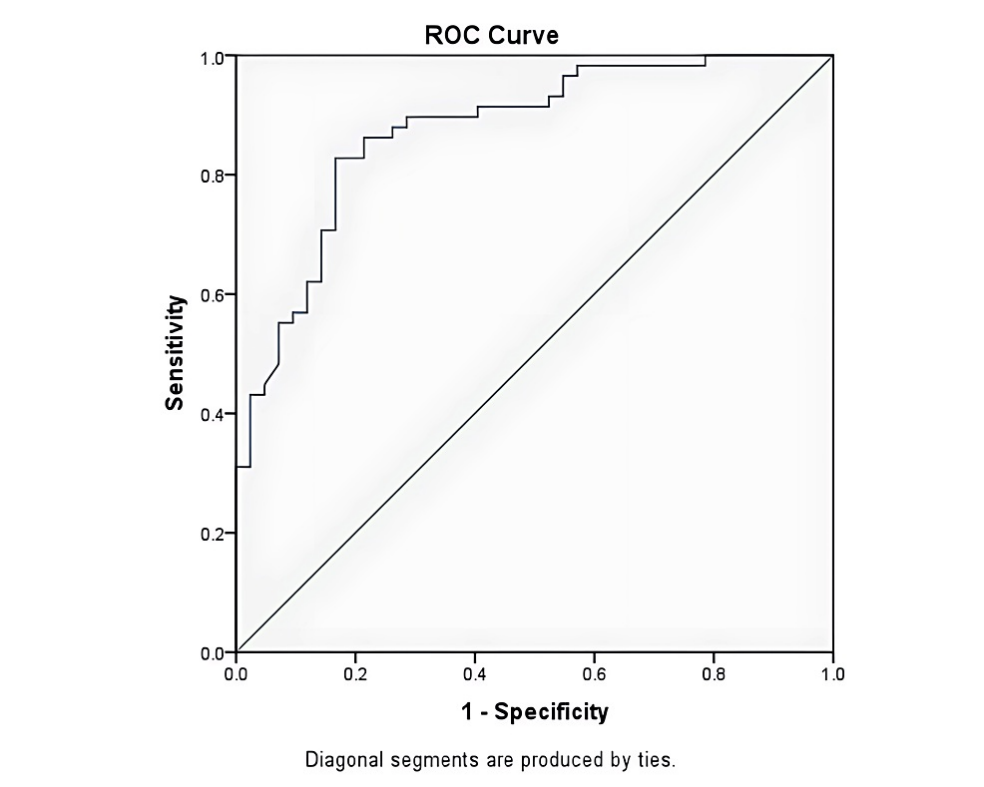
Receiver operating characteristic curve of the tumor contact length measured in the axial plane. ROC: Receiver operating characteristic

The current literature reveals a dearth of studies examining the role of tumor height, tumor contact length, and the H/CL ratio using the CT method in bladder cancers. Existing studies employing CT for grading typically involve radiomics studies derived from contrast-enhanced imaging. In the CT-based radiomics investigation conducted by Zhang et al. in 2020, a comprehensive exploration of tumor morphology and tissue patterns was undertaken to discriminate between high-grade BC and low-grade BC [19]. The AUC in the study group reached a noteworthy 0.950 (95% CI, 0.912-0.988), with a slightly reduced AUC of 0.860 (95% CI, 0.742-0.979) in the validation group. Notably, diagnostic parameters exhibited robust performance, with an accuracy of 83.8%, specificity of 88.5%, sensitivity of 72.7%, positive predictive value of 88.5%, and negative predictive value of 72.7%. Utilizing our ROC curve analysis in our study, contact length values surpassing 19.1mm were found to discriminate high-grade tumors from low-grade tumors (Figure 4). In this context, using 19.1mm cutoff value for tumor contact length, high-grade tumors and low-grade tumors could be distinguished with 83% sensitivity, 83% specificity, 83% negative predictive value, and 83% accuracy. Parallel diagnostic metrics, including specificity, sensitivity, and both positive and negative predictive values, were observed in our study, aligning with the outcomes of Zhang et al. [19]. This underscores the consistency and reliability of these parameters across different investigations. Remarkably, our study delved into the utility of CL measured in the axial plane via CT urography for detecting high-grade bladder tumors. Comparatively, our study introduces a novel perspective by focusing on morphological features rather than atomic properties for grading.

In a study conducted by Bovenkamp et al., 93% accuracy was reported in discriminating between low-grade and high-grade bladder tumors. This discrimination was achieved through the application of optical coherence CT and spectroscopy [20]. However, it is pivotal to note that the effectiveness of this study lies in its ability to predict grading using spectroscopic techniques, prioritizing chemical attributes over the exclusive reliance on morphological features of the tumor. Similarly, in a study employing MRI, the reported diffusion coefficient observed in synthetic MRI exhibited a specificity of 77.8% and sensitivity of 87.9% in the detection of high-grade bladder tumors [21]. In consonance with the spectroscopy study, this investigation relies on discerning disparities in proton movement within the tumor tissue, emphasizing chemical properties rather than solely relying on morphological data for diagnostic purposes.

Another promising area for investigation is artificial intelligence (AI). BC, which is widely prevalent and lacks routine screening, represents an ideal candidate for the application of emerging AI techniques. CADe enhances sensitivity by identifying subtle lesions, while CADx enhances specificity by discriminating between benign and malignant findings, utilizing computer-assisted detection and diagnosis, respectively [22]. AI has shown success in segmenting bladder tumors and monitoring treatment response [23]. However, there is a paucity of research exploring its prospective use in detecting incidental bladder tumors and characterizing lesions, indicating the need for additional investigation in this area [24].

Recognizing and addressing the inherent limitations of this study is crucial for a thorough understanding of its scope and potential implications. The concentration on a single-center approach, while advantageous for in-depth exploration, raises considerations about the broader applicability of the findings. The relatively modest sample size, consisting of 100 patients, underscores the necessity for validation across larger, multi-center cohorts to fortify the reliability and generalizability of the study's outcomes. The retrospective nature of the design, though valuable for leveraging existing data, introduces the potential for selection bias, underscoring the significance of future prospective studies to validate and extend the observed associations. Moreover, the intricacies associated with confirming histopathological correspondence in cases with multiple localized tumors present challenges to result interpretation. A prospective methodology, coupled with meticulous coordination between radiological and histopathological assessments, would enhance result accuracy and reliability. Additionally, while the study's focus on CT imaging provides valuable insights, a more comprehensive understanding of bladder cancer characteristics may be achieved by integrating other imaging modalities or biomarkers. Transparent acknowledgment of these limitations is pivotal, laying the groundwork for future research endeavors. Efforts to address these challenges through collaborative, multi-center investigations and prospective study designs will bolster the scientific rigor and significance of subsequent studies in this field.

Conversely, the study’s strengths lie in its pioneering approach, as the tumor has not been previously graded based on morphological features in imaging. Unlike prior studies attempting to differentiate tumor grading based on variations in chemical and atomic properties, this study provides novel insights into the relationship between CT-derived numerical measurements, including tumor morphology, H, CL, H/CL ratio, and tumor grade.

In the scope of CT, a notable correlation emerges between tumor characteristics and grade, encompassing morphology, H, CL, and the H/CL ratio. These quantitative measurements stand poised as valuable adjunctive criteria for preoperative predictions of tumor grade, enhancing our understanding of morphological determinants in the context of bladder cancer. Future research avenues may explore the integration of additional imaging modalities or consider advancements in machine learning techniques to enhance the predictive capabilities of quantitative CT analysis.

## Conclusions

In conclusion, our study underscores the efficacy of CT as a robust tool for preoperatively assessing the tumor grade in BC. The observed correlation between the CL of the tumor and the distended bladder wall in preoperative abdominopelvic CT indicates a promising role for CT in grading BC before surgical intervention. Notably, an increase in the tumor CL aligns with a heightened probability of a high-grade tumor. Leveraging this predictive capacity in preoperative evaluations is poised to significantly enhance the precision of treatment planning, thereby optimizing therapeutic strategies for patients diagnosed with BC.
